# Supplementary material for: Antimicrobial Resistance in Escherichia coli and Its Correlation with Antimicrobial Use on Commercial Poultry Farms in Bangladesh
Source: Antibiotics (Basel). 2023 Aug 24;12(9):1361. doi: 10.3390/antibiotics12091361 (PMC10525429; doi:10.3390/antibiotics12091361)
Supplement: Supplementary file 1 [file antibiotics-12-01361-s001.zip › antibiotics-2543633-supplementary.pdf]

Table S1: Minimum inhibitory concentration distribution for *E. coli* in fecal samples of broiler

Antimicrobial agent	Number of strains with MIC (mg/ml)																								No. of isolates
															Susceptible		Intermediate		Resistant		Wild type		Non-wild type		
	≤0.03	0.06	0.12	0.25	0.5	1	2	4	8	16	32	64	128	256	[n]	[%]	[n]	[%]	[n]	[%]	[n]	[%]	[n]	[%]	
Gentamicin					*43	156	119	24	2	1	5	89			318	72	26	6	95	22	318	72	121	28	439
Tetracycline							*1	6	4		4	44	380		1	0	10	2	428	98	11	3	428	97	439
Ceftazidime					*415	4	2	2	**8**	8					421	96	10	2	8	2	419	95	20	5	439
Trimethoprim				*12	6	3	1					417			22	5	0	0	417	95	22	5	417	95	439
Ampicillin							13	17				3	406		30	7	0	0	409	93	30	7	409	93	439
Chloramphenicol									*73	98	58	60	50	100	0	0	171	39	268	61	171	39	268	61	439
Azithromycin									30	139	71	48	151		30	7	139	31	270	62	169	38	270	62	439
Nalidixic acid								*9	13	**25**	2	5	6	379	22	5	25	6	392	89	22	5	417	95	439
Colistin						*377	3	24	34	1					377	86	3	1	59	13	380	87	59	13	439
Cefotaxime				*417	3	2	4	1	12						420	96	6	1	13	3	417	95	22	5	439
Meropenem	*439														439	100	0	0	0	0	439	100	0	0	439
Ciprofloxacin	*7			2	27	**18**	10	18	19	338					7	2	29	6	403	92	7	2	432	98	439
Tigecycline				*201	136	79	17	2	1	3					-	-	-	-	-	-	337	77	102	23	439

Antimicrobial agent	Number of strains with MIC (mg/ml)																			No. of isolates
										Susceptible		Intermediate		Resistant		Wild type		Non-wild type		
	≤8	16	32	64	128	256	512	1024	2048	[n]	[%]	[n]	[%]	[n]	[%]	[n]	[%]	[n]	[%]	
Sulfamethaxole	*8	7	6	5					413	26	6	0	0	413	94	26	6	413	94	439

* No visible growth at this concentration, meaning MIC is equal to or below this concentration. | -- CLSI breakpoint between susceptible and intermediate, | -- CLSI breakpoint between intermediate and resistant and | -- ECOFF value between wild type and non-wild type.

Table S2: Minimum inhibitory concentration distribution for *E. coli* in environmental samples of broiler

**Antimicrobial agent**	**Number of strains with MIC (mg/ml)**																										**No. of isolates**
															**Susceptible**		**Intermediate**		**Resistant**		**Wild type**		**Non-wild type**		
	**≤0.03**	**0.06**	**0.12**	**0.25**	**0.5**	**1**	**2**	**4**	**8**	**16**	**32**	**64**	**128**	**256**	**[n]**	**[%]**	**[n]**	**[%]**	**[n]**	**[%]**	**[n]**	**[%]**	**[n]**	**[%]**	
Gentamicin					*3	52	26	3	1		1	11			81	84	4	4	12	12	81	84	16	16	97
Tetracycline							*1	1			1	14	80		1	1	1	1	95	98	2	2	95	98	97
Ceftazidime					*89	4	4								97	100	0	0	0	0	93	96	4	4	97
Trimethoprim				*17	3	1						76			21	22	0	0	76	78	21	22	76	78	97
Ampicillin							5	6		3		3	80		11	11	3	3	83	86	11	11	86	89	97
Chloramphenicol									*28	36	3	12	7	11	0	0	64	66	33	34	64	66	33	34	97
Azithromycin									4	28	17	12	36		4	4	28	29	65	67	32	33	65	67	97
Nalidixic acid								*11	13	8	3		1	61	24	25	8	8	65	67	24	25	73	75	97
Colistin						*89	2	1	1	2	2				89	92	2	2	6	6	91	94	6	6	97
Cefotaxime				*89	6	1			1						95	98	1	1	1	1	89	92	8	8	97
Meropenem	*97														97	100	0	0	0	0	97	100	0	0	97
Ciprofloxacin	*6		1	6	17	5	2	6	5	49					7	7	23	24	67	69	6	6	91	94	97
Tigecycline				*29	43	22	3								-	-	-	-	-	-	72	74	25	26	97

**Antimicrobial agent**	**Number of strains with MIC (mg/ml)**																			**No. of isolates**
										**Susceptible**		**Intermediate**		**Resistant**		**Wild type**		**Non-wild type**		
	**≤8**	**16**	**32**	**64**	**128**	**256**	**512**	**1024**	**2048**	**[n]**	**[%]**	**[n]**	**[%]**	**[n]**	**[%]**	**[n]**	**[%]**	**[n]**	**[%]**	
Sulfamethaxole	*8	4	2	1					82	15	15	0	0	82	85	15	15	82	85	97

* No visible growth at this concentration, meaning MIC is equal to or below this concentration. -- CLSI breakpoint between susceptible and intermediate, -- CLSI breakpoint between intermediate and resistant and -- ECOFF value between wild type and non-wild type.

Table S3: Minimum inhibitory concentration distribution for *E. coli* in fecal samples of Sonali

Antimicrobial agent	Number of strains with MIC (mg/ml)														Susceptible		Intermediate		Resistant		Wild type		Non-wild type		No. of isolates
	≤0.03	0.06	0.12	0.25	0.5	1	2	4	8	16	32	64	128	256	[n]	[%]	[n]	[%]	[n]	[%]	[n]	[%]	[n]	[%]	
Gentamicin					*24	75	81	14	1			57			180	71	15	6	57	23	180	71	72	29	252
Tetracycline							*1				2	25	224		1	0	0	0	251	100	1	0	251	100	252
Ceftazidime					*241	1	2	1	3	4					244	96	4	2	4	2	242	96	11	4	252
Trimethoprim				*6	5	3		2			1	235			16	6	0	0	236	94	14	6	238	94	252
Ampicillin						*1	8	8	2	1			232		17	7	3	1	232	92	19	8	233	92	252
Chloramphenicol									36	40	15	29	28	104	0	0	76	30	176	70	76	30	176	70	252
Azithromycin								1	7	62	33	29	120		8	3	62	25	182	72	70	28	182	72	252
Nalidixic acid								*1	4	15	7	3		222	5	2	15	6	232	92	5	2	247	98	252
Colistin						*227	4	10	10		1				227	90	4	2	21	8	231	92	21	8	252
Cefotaxime				*238	5		3		6						243	97	3	1	6	2	238	94	14	6	252
Meropenem		*252													252	100	0	0	0	0	252	100	0	0	252
Ciprofloxacin	*1	1	2		11	**11**	13	9	20	184					4	2	11	4	237	94	2	1	250	99	252
Tigecycline				*73	94	68	8	2	3	4					-	-	-	-	-	-	167	66	85	34	252

Antimicrobial agent	Number of strains with MIC (mg/ml)									Susceptible		Intermediate		Resistant		Wild type		Non-wild type		No. of isolates
	≤8	16	32	64	128	256	512	1024	2048	[n]	[%]	[n]	[%]	[n]	[%]	[n]	[%]	[n]	[%]	
Sulfamethaxole	2	4	5					1	240	11	4	0	0	241	96	11	4	241	96	252

* No visible growth at this concentration, meaning MIC is equal to or below this concentration. (green bar) -- CLSI breakpoint between susceptible and intermediate, (red bar) -- CLSI breakpoint between intermediate and resistant and (black bar) -- ECOFF value between wild type and non-wild type.

Table S4: Minimum inhibitory concentration distribution for *E. coli* in environmental samples of Sonali

Antimicrobial agent	Number of strains with MIC (mg/ml)														Susceptible		Intermediate		Resistant		Wild type		Non- wild type		No. of isolates
	≤0.03	0.06	0.12	0.25	0.5	1	2	4	8	16	32	64	128	256	[n]	[%]	[n]	[%]	[n]	[%]	[n]	[%]	[n]	[%]	
Gentamicin					*9	20	22	5			1	9			51	77	5	8	10	15	51	77	15	23	66
Tetracycline							*3	5	2	3		5	48		3	5	7	10	56	85	10	15	56	85	66
Ceftazidime					*63	1	1			1					65	98	0	0	1	2	64	97	2	3	66
Trimethorim				*4	10	3						49			17	26	0	0	49	74	17	26	49	74	66
Ampicilline							7	7	1		2	1	48		14	21	1	2	51	77	15	23	51	77	66
Chloramphenicol									*22	23	1	3		17	0	0	45	68	21	32	45	68	21	32	66
Azithromycin									1	21	11	16	17		1	2	21	32	44	67	22	33	44	67	66
Nalidixic acid								*12	9	4	3		1	37	21	32	4	6	41	62	21	32	45	68	66
Colistin						*54	2	1	5		4				54	82	2	3	10	15	56	85	10	15	66
Cefotaxime				*63	1		2								64	97	2	3	0	0	63	95	3	5	66
Meropenem		*66													66	100	0	0	0	0	66	100	0	0	66
Ciprofloxacin	*7	3	1	2	10	3	2	4	1	33					11	17	12	18	43	65	10	15	56	85	66
Tigecycline				*16	30	10	5	4	1						-	-	-	-	-	-	46	70	20	30	66

Antimicrobial agent	Number of strains with MIC (mg/ml)									Susceptible		Intermediate		Resistant		Wild type		Non-wild type		No. of isolates
	≤8	16	32	64	128	256	512	1024	2048	[n]	[%]	[n]	[%]	[n]	[%]	[n]	[%]	[n]	[%]	
Sulfamethaxole	1	3	7	4	2				49	17	26	0	0	49	74	17	26	49	74	66

* No visible growth at this concentration, meaning MIC is equal to or below this concentration. -- CLSI breakpoint between susceptible and intermediate, -- CLSI breakpoint between intermediate and resistant and -- ECOFF value between wild type and non-wild type
